# Supplementary figures and images for: Production and сharacterization of the exopolysaccharide from strain Paenibacillus polymyxa 2020
Source: PLoS One. 2021 Jul 6;16(7):e0253482. doi: 10.1371/journal.pone.0253482 (PMC8259973; doi:10.1371/journal.pone.0253482)

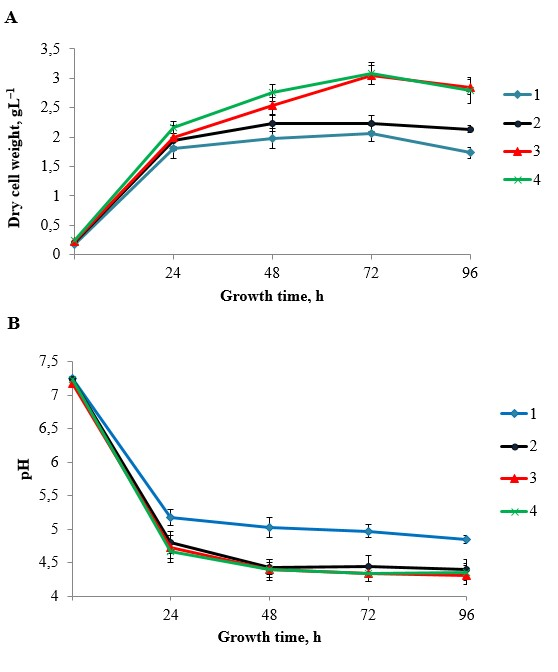

Supplement: S1 Fig — Biomass accumulation (A) and the changes in pH (B) during growth of P. polymyxa 2020 in a sucrose medium containing sucrose at concentrations 50 g L-1 (1), 100 g L-1 (2), 150 g L-1 (3), 200 g L-1 (4). (TIF) [file pone.0253482.s001.tif]

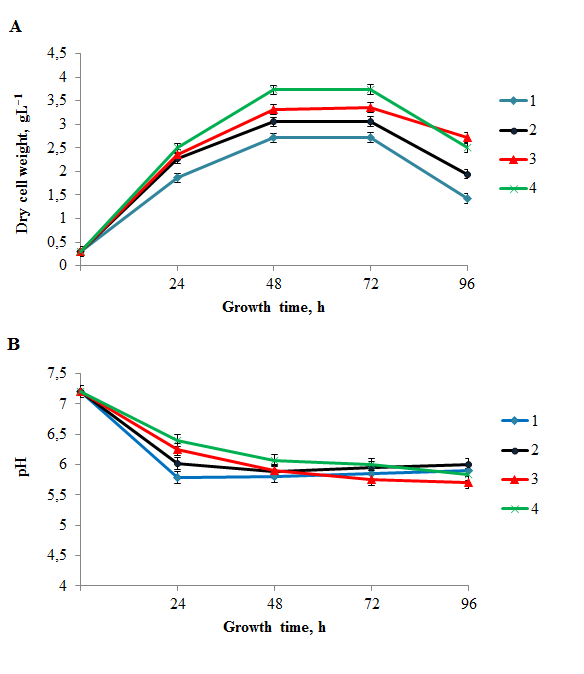

Supplement: S2 Fig — Biomass accumulation (A) and the changes in pH (B) during growth of P. polymyxa 2020 in molasses medium containing sucrose at concentrations 50 g L-1 (1), 100 g L-1 (2), 150 g L-1 (3), 200 g L-1 (4). (TIF) [file pone.0253482.s002.tif]

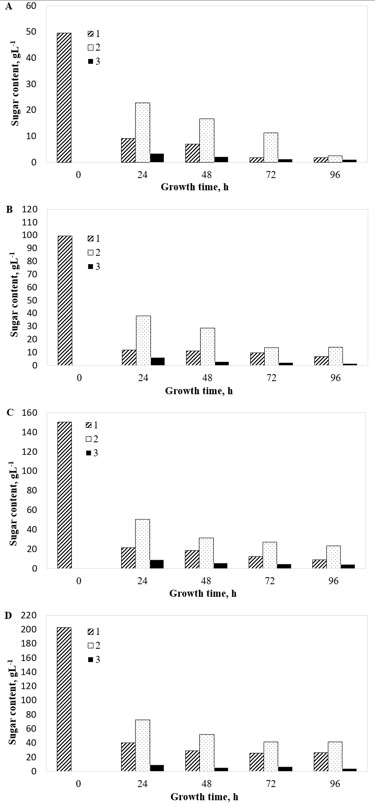

Supplement: S3 Fig — Sucrose (1), glucose (2) and fructose (3) content during growth of P. polymyxa 2020 in a culture medium with sucrose at an initial concentration of 50 g L-1 (A), 100 g L-1 (B), 150 g L-1 (C) and 200 g L-1 (D) for 4 days (by HPLC). (TIF) [file pone.0253482.s003.tif]

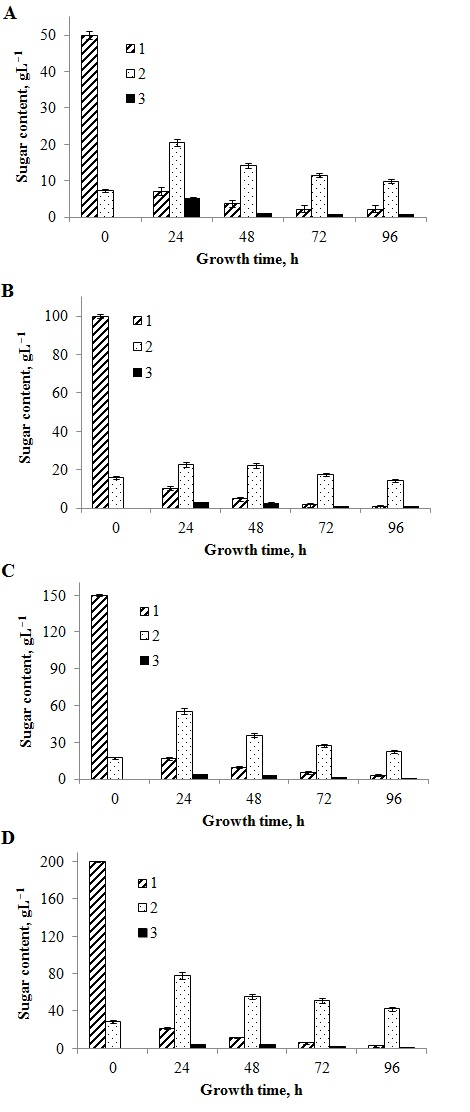

Supplement: S4 Fig — Sucrose (1), glucose (2) and fructose (3) content during growth of P. polymyxa 2020 in a molasses medium with sucrose at an initial concentration of 50 g L-1 (A), 100 g L-1 (B), 150 g L-1 (C) and 200 g L-1 (D) for 4 days (by HPLC). (TIF) [file pone.0253482.s004.tif]

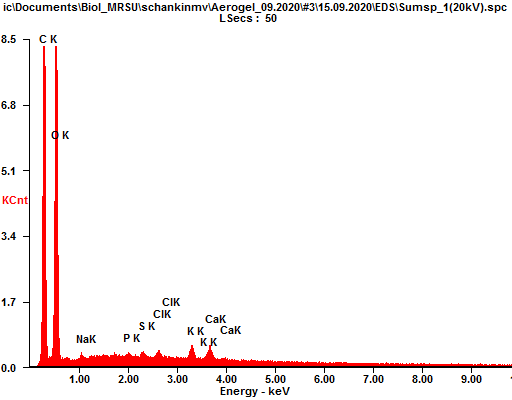


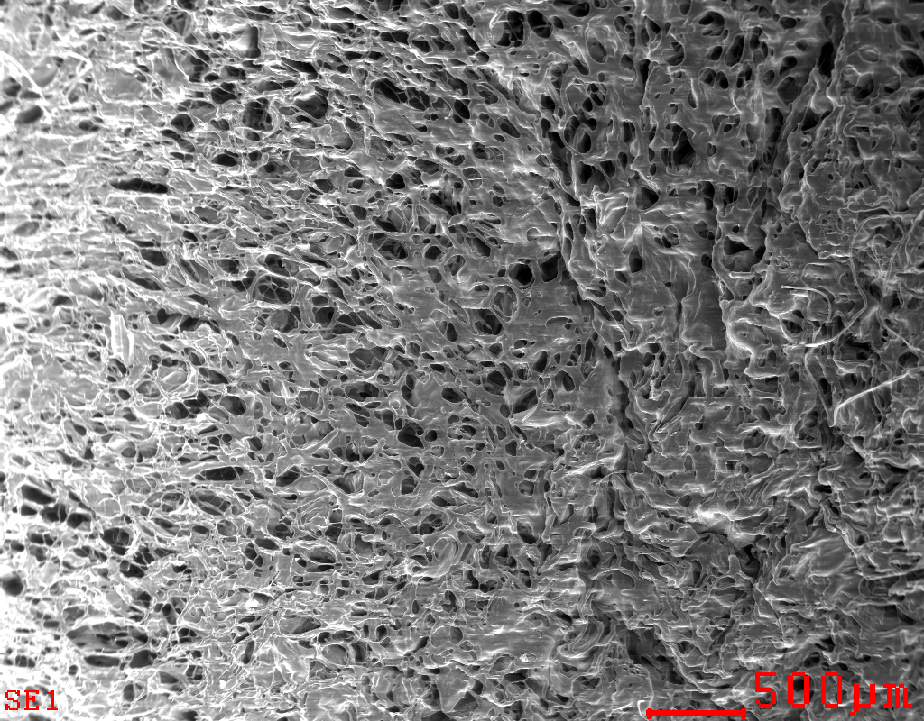


| ***Element*** | ***Wt%*** | ***At%*** |
| --- | --- | --- |
| ***CK*** | 49.86 | 57.49 |
| ***OK*** | 48.25 | 41.77 |
| ***NaK*** | 00.21 | 00.13 |
| ***PK*** | 00.10 | 00.04 |
| ***SK*** | 00.14 | 00.06 |
| ***ClK*** | 00.23 | 00.09 |
| ***KK*** | 00.55 | 00.19 |
| ***CaK*** | 00.66 | 00.23 |
| ***Matrix*** | Correction | ZAF |

Supplement: S5 Fig — (DOC) [file pone.0253482.s005.doc]

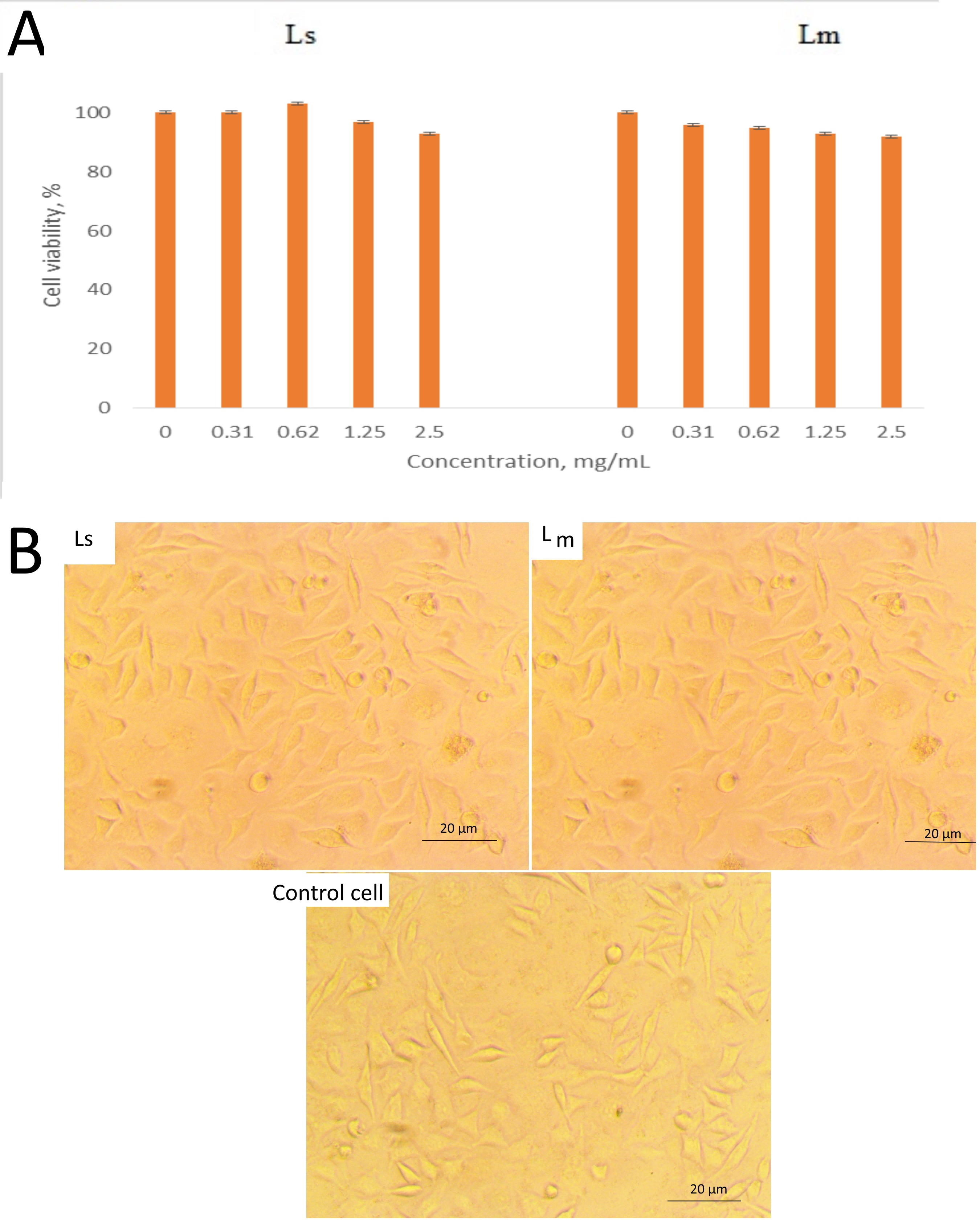

Supplement: S6 Fig — Cell viabilities (A) and cell morphologies (B) of the mouse fibroblast cell culture L929 treated with levan produced P. polymyxa 2020 in a sucrose (Ls) and molasses (Lm) media. (TIF) [file pone.0253482.s006.tif]

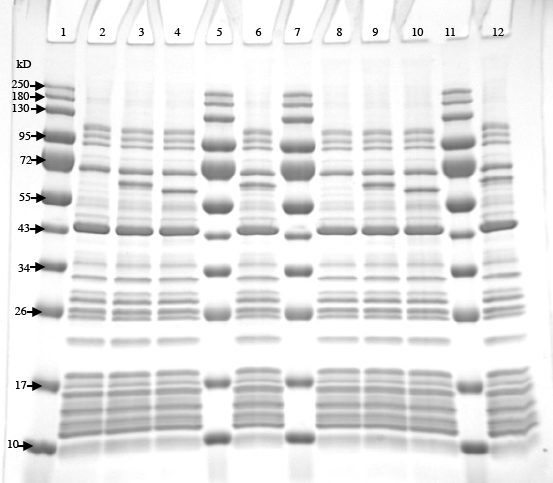

Supplement: S7 Fig — Lanes 1, 5, 7, 11–10 μl of color prestained protein standards broad range (10-250kDa) (NEB #P7719); lanes 2, 8–2.5 μl of PurExpress extract from empty vector pSAPv6; lanes 3, 9–2.5 μl of PurExpress extracts from pPpo2020_sacB template plasmid; lanes 4, 10–2.5 μl of PurExpress extracts from pPpo2020_sacC template plasmid; lanes 6, 12–2.5 μl of PurExpress extracts from pPpo2020_sacBC template plasmid; plasmids purified from ER2683 E.coli strain (lanes: 2, 3, 4, 6); plasmids purified from ER3081 E.coli strain (lanes 8, 9, 10, 12). (TIF) [file pone.0253482.s007.tif]
