# Supplementary material for: Production and сharacterization of the exopolysaccharide from strain Paenibacillus polymyxa 2020
Source: PLoS One. 2021 Jul 6;16(7):e0253482. doi: 10.1371/journal.pone.0253482 (PMC8259973; doi:10.1371/journal.pone.0253482)
Supplement: Attachment — Submitted filename: Response letter edits(3).docx [file pone.0253482.s009.docx]

PONE-D-20-40095
Production and сharacterization of the exopolysaccharide from strain Paenibacillus polymyxa 2020
PLOS ONE

1.Please ensure that your manuscript meets PLOS ONE's style requirements, including those for file naming. The PLOS ONE style templates can be found at

https://journals.plos.org/plosone/s/file?id=wjVg/PLOSOne_formatting_sample_main_body.pdf and

<https://journals.plos.org/plosone/s/file?id=ba62/PLOSOne_formatting_sample_title_authors_affiliations.pdf>

**Authors’ response**: Files were reformatted according to PLOS One style.

2.We note that you have indicated that data from this study are available upon request. PLOS only allows data to be available upon request if there are legal or ethical restrictions on sharing data publicly. For more information on unacceptable data access restrictions, please see <http://journals.plos.org/plosone/s/data-availability#loc-unacceptable-data-access-restrictions>.

In your revised cover letter, please address the following prompts:
 
a) If there are ethical or legal restrictions on sharing a de-identified data set, please explain them in detail (e.g., data contain potentially sensitive information, data are owned by a third-party organization, etc.) and who has imposed them (e.g., an ethics committee). Please also provide contact information for a data access committee, ethics committee, or other institutional body to which data requests may be sent.
 
b) If there are no restrictions, please upload the minimal anonymized data set necessary to replicate your study findings as either Supporting Information files or to a stable, public repository and provide us with the relevant URLs, DOIs, or accession numbers. For a list of acceptable repositories, please see http://journals.plos.org/plosone/s/data-availability#loc-recommended-repositories.
 
We will update your Data Availability statement on your behalf to reflect the information you provide.

**Authors’ response**: All appropriate data were deposited into the NCBI database. Lines 315-321 have all appropriate references. There are no restrictions on the data.

3. We note that the grant information you provided in the ‘Funding Information’ and ‘Financial Disclosure’ sections do not match.

**Authors’ response**:

This work was supported by the Ministry of Science and Higher Education of the Russian Federation, grant number FZRS-2020-0003. New England Biolabs provided support in the form of salaries for authors A.F, S.V. and R.J.R. The specific roles of these authors are articulated in the ‘author contributions’ section. The funders had no role in study design, data collection and analysis, decision to publish, or preparation of the manuscript.'

Please remove second reference to grant 18-14-00098 from a system, because I cannot update it

"The instrumental and computational NMR investigation was carried out within the Glycoinformatics project funded by Russian Science Foundation, grant 18-14-00098".

4. Please upload a copy of Figure 14, to which you refer in your text on page 22. If the figure is no longer to be included as part of the submission please remove all reference to it within the text.

**Authors' response:** All figures were uploaded. Their numbering was changed. New figures were added.

5.Thank you for stating the following in the Competing Interests section:
"A.F, S.V. and R.J.R. work for New England Biolabs, company that sells research reagents, including restriction, glyco enzymes and DNA methyltransferases, to the scientific community. "
We note that one or more of the authors are employed by a commercial company: New England Biolabs Inc.,
 
a) Please provide an amended Funding Statement declaring this commercial affiliation, as well as a statement regarding the Role of Funders in your study. If the funding organization did not play a role in the study design, data collection and analysis, decision to publish, or preparation of the manuscript and only provided financial support in the form of authors' salaries and/or research materials, please review your statements relating to the author contributions, and ensure you have specifically and accurately indicated the role(s) that these authors had in your study. You can update author roles in the Author Contributions section of the online submission form.
 
Please also include the following statement within your amended Funding Statement.
“The funder provided support in the form of salaries for authors [insert relevant initials], but did not have any additional role in the study design, data collection and analysis, decision to publish, or preparation of the manuscript. The specific roles of these authors are articulated in the ‘author contributions’ section.”
If your commercial affiliation did play a role in your study, please state and explain this role within your updated Funding Statement.
 
b) Please also provide an updated Competing Interests Statement declaring this commercial affiliation along with any other relevant declarations relating to employment, consultancy, patents, products in development, or marketed products, etc.  
 
Within your Competing Interests Statement, please confirm that this commercial affiliation does not alter your adherence to all PLOS ONE policies on sharing data and materials by including the following statement: "This does not alter our adherence to PLOS ONE policies on sharing data and materials.” (as detailed online in our guide for authors [http://journals.plos.org/plosone/s/competing-interests](about:blank)) . If this adherence statement is not accurate and there are restrictions on sharing of data and/or materials, please state these. Please note that we cannot proceed with consideration of your article until this information has been declared.
 
Please include both an updated Funding Statement and Competing Interests Statement in your cover letter. We will change the online submission form on your behalf.
 
Please know it is PLOS ONE policy for corresponding authors to declare, on behalf of all authors, all potential competing interests for the purposes of transparency. PLOS defines a competing interest as anything that interferes with, or could reasonably be perceived as interfering with, the full and objective presentation, peer review, editorial decision-making, or publication of research or non-research articles submitted to one of the journals. Competing interests can be financial or non-financial, professional, or personal. Competing interests can arise in relationship to an organization or another person. Please follow this link to our website for more details on competing interests: [http://journals.plos.org/plosone/s/competing-interests](about:blank)

**Authors' response:**

The authors have read the journal's policy and have the following competing interests: A.F, S.V. and R.J.R. are paid employees of New England Biolabs (<https://www.neb.com/>). There are no patents, products in development or marketed products associated with this research to declare. This does not alter our adherence to PLOS ONE policies on sharing data and materials.'

6.Thank you for submitting the above manuscript to PLOS ONE. During our internal evaluation of the manuscript, we found significant text overlap between your submission and the following previously published works, some of which you are an author.

Introduction section:
- <https://www.sciencedirect.com/science/article/abs/pii/S0141813018332847?via%3Dihub>

Results, Discussion, and Conclusion sections:
- <https://microbialcellfactories.biomedcentral.com/articles/10.1186/s12934-016-0603-7>
- <https://mra.asm.org/content/8/32/e00657-19>
- <https://pubmed.ncbi.nlm.nih.gov/31879785/>
- <https://www.sciencedirect.com/science/article/abs/pii/S0141813018332847?via%3Dihub>
- <https://link.springer.com/article/10.1007/BF01569633>
- <https://www.sciencedirect.com/science/article/abs/pii/S0141813016308777?via%3Dihub>
- <https://www.sciencedirect.com/science/article/abs/pii/S014486171630114X?via%3Dihub>
- <https://www.frontiersin.org/articles/10.3389/fbioe.2020.603407/full>

We would like to make you aware that copying extracts from previous publications, especially outside the methods section, word-for-word is unacceptable. In addition, the reproduction of text from published reports has implications for the copyright that may apply to the publications.

Please revise the manuscript to rephrase the duplicated text, cite your sources, and provide details as to how the current manuscript advances on previous work. Please note that further consideration is dependent on the submission of a manuscript that addresses these concerns about the overlap in text with published work.

We will carefully review your manuscript upon resubmission, so please ensure that your revision is thorough.

**Authors' response:**

I would like to apologize for the presence of duplicated paragraphs in our original submission. The original manuscript was written in Russian with small pieces of paragraphs written in English. While making the full translation I did not realize that these pieces were merely exact language taken from cited articles. We rephrased these duplicated paragraphs as shown below.

**The duplicated text from Grady et al., 2016. Revised to Line 281-283**

Members of the *Paenibacillus* genus have been isolated from different environments. While many of the species are relevant to humans, animals, and plants, the majority are found in soil, often associated with plant roots to promote growth They can be to improve agriculture [31].

**The duplicated text from Revin et al., 2020. Revised to Line 228-242**

The L929 mouse fibroblast cell culture used in this study was obtained from the tissue culture collection of the D.I. Ivanovsky Institute of Virology, Russia. The cells were cultured in Dulbecco’s modified Eagle's medium (DMEM) (Paneko, Russia) containing 10% fetal bovine serum (FBS) (HyClone, USA) in the presence of penicillin and streptomycin under standard conditions: 5% CO_2_ atmosphere, t = 37°C, 5% humidity in an MCO-170M incubator (Sanyo, Japan). Cells in exponential growth phase were dispened into a 96-well plate (5 × 10^3^ cells/well). After 24 hr, fresh medium, containing the EPSs was added to the 96 wells and incubated for a further 24hr. Wells without EPSs were used as a control. The morphological structure of the cells were monitored using an inverted optical microscope (Micromed, Russia). The MTT assay was used to measure cytotoxicity of the EPSs. The medium was replaced with a fresh one containing 5 mg/mL dimethyl thiazolyl diphenyl (MTT). After 4 h incubation time the medium was removed and 150 μL DMSO was added. The optical density was measured on a microplate reader EFOS 9305 (Russia) at a wavelength of 492 nm with a reference wavelength of 620 nm. Cell viability was recorded as the ratio of the optical density of the sample to the control and expressed as a percentage.

**The duplicated text from H.Mankai et.al., 2019. Revised to Line 151-164**

SMRTbell libraries were prepared using a modified PacBio protocol adapted for NEB reagents. Genomic DNA samples were sheared to an average size of ~ 6-10 kb using the G-tube protocol (Covaris; Woburn, MA, USA), treated with FFPE, end repaired, and ligated with hairpin adapters. Incompletely formed SMRTbell templates were removed by digestion with a combination of exonuclease III and exonuclease VII (New England Biolabs; Ipswich, MA, USA). The qualification and quantification of the SMRTbell libraries were made on a Qubit fluorimeter (Invitrogen, Eugene, OR) and a 2100 Bioanalyzer (Agilent Technologies, Santa Clara, CA). SMRT sequencing was performed using a PacBio RSII (Pacific Biosciences; Menlo Park, CA, USA) based on standard protocols for 6-10 kb SMRTbell library inserts. Sequencing reads were collected and processed using the SMRT Analysis pipeline from Pacific Biosciences

(http://www.pacbiodevnet.com/SMRT-Analysis/Software/SMRT-Pipe) [53].

Next-generation SMRT sequencing technology from Pacific Biosciences Inc. allowed the assembly of a complete circular genome. It also enabled the determination of the m6A and m4C modified motifs. [54-56].

**The duplicated text from Grady et al., 2016. Revised to Line 323-329**

The genome sequences of 212 strains of *Paenibacillus*, that represent 82 different species are available [31]. Genome sizes range from 3.02 Mb for *P. darwinianus* Br, isolated from Antarctic soil [60] to 8.82 Mb for *P. mucilaginosus* K02, implicated in silicate mineral weathering [61] and the number of genes varies from 3,064 for *P. darwinianus* Br to 8,478 *P. sophorae* S27. The insect pathogens *P. darwinianus*, *P. larvae* and *P. popilliae* have smaller genomes from 4.51 and 3.83 Mb, respectively, perhaps reflecting their niche specialization. The GC content of *Paenibacillus* DNA ranges from 39 to 59 % [62].

**The duplicated text from J.R Horton et al., 2020. Revised to Line 285-322**

A 6 kb SMRTbell library was sequenced using C4-P6 chemistry and run on 4 SMRT cells with a 360-minute collection time. 1.6 Gb of data were collected from 55,864 sequencing reads with 1,705 bp mean subread lengths. This sequence was assembled *de novo* using the HGAP_Assembly.3 version 2.3.0 with default quality and read length parameters and polished once using Quiver. The original polished assembly generated 125 linear contigs (the sum of the contigs equaled 5,907,830 bp) with N50 equal to 104,550bp. BLAST analysis at NCBI of the 125 contig assembly of *Paenibacillus polymyxa* 2020 identified the strongest hits as having 99.5% homology to [AFOX01](https://www.ncbi.nlm.nih.gov/nuccore/AFOX00000000.1/) a shotgun Miseq-based sequence assembly of *P. polymyxa* DSM36/ATCC842 [42].

*P. polymyxa* DSM36 native genomic DNA (2μg) was obtained directly from the DSMZ collection and a 15kb SMRT bell library was prepared according to the Pacific Biosciences 10-20 kb protocol including additional separation using a Blue Pippin and sequenced with C4-P6 chemistry on 2 SMRT cells. One library consisted of non-size selected (15 kb) DNA and one contained size-selected (17kb) DNA and a 360-minute collection time was used for each sample on the RSII PacBio sequencer. 28,951 sequencing reads with 12,000 bp mean subread lengths, yielded 0.35 Gb of data. This was *de novo* assembled using the HGAP_Assembly.3 version 2.3.0 with default quality and read length parameters and polished 3 times using Quiver. The polished assembly generated 2 closed circular genomic elements of 5,919,686 bp with 45.08 % GC content for the main chromosome and 45,518 bp with 41.76% GC for the plasmid pPpo45. The assembled sequences were submitted to the NCBI Prokaryotic Genomes Annotation Pipeline (PGAP) [57, 58].

A re-run of the original reads from *P. polymyxa* 2020 against the genomic reference sequence of *P. polymyxa* DSM36 and polishing 5 times using Quiver demonstrated that 97.7% of the *P. polymyxa* 2020 reads mapped to the *P. polymyxa* DSM36 reference and generated a final genome with 99.99 % homology between the two strains.

The same m6A modified DNA motif, CN**A**GNNNNNTTGK, was detected in both *P. polymyxa* 2020 and DSM36 strains and can be assigned to a Type I restriction-modification system Ppo36I in the DSM36 strain and Ppo2020I in the 2020 strain (**Fig 1**), consistent with the notion that these two *P. polymyxa* strains are essentially identical. All data have been deposited at NCBI and in REBASE [59].

**Nucleotide sequence accession numbers**

The complete genome sequence of *P. polymyxa* DSM36 was deposited in NCBI and given the accession numbers: CP049783-CP049784. The original sequence reads also have been deposited at NCBI under SRA [SRR11271650](https://dataview.ncbi.nlm.nih.gov/object/SRR11271650) and [SRR11271651](https://dataview.ncbi.nlm.nih.gov/object/SRR11271651).Biosample : [SAMN14247784](https://dataview.ncbi.nlm.nih.gov/object/SAMN14247784). The complete genome sequence of *P. polymyxa* 2020 also is available in GenBank with the accession numbers: CP049598-CP049599. The original sequence reads have been deposited at NCBI under SRA: [SRR11236808](https://dataview.ncbi.nlm.nih.gov/object/SRR11236808); [SRR11236809](https://dataview.ncbi.nlm.nih.gov/object/SRR11236809); [SRR11236810](https://dataview.ncbi.nlm.nih.gov/object/SRR11236810); [SRR11236811](https://dataview.ncbi.nlm.nih.gov/object/SRR11236811). Biosample: SAMN14247689. Data for both strains are available under Bioproject: [PRJNA609344](https://dataview.ncbi.nlm.nih.gov/object/PRJNA609344)

**The duplicated text from Gojgic-Cvijovic et al., 2018. Revised to Line 348-352**

The use of sugar-based raw materials as nutritive substances can greatly help to reduce the production cost of microbial polysaccharides [49]. Molasses, a food industry by-product of cane or beet sugar production, contains up to 80% of dry matter, of which sucrose represents ~48%. Molasses also contains amino acids, organic acids and their salts, betaine, mineral compounds, and vitamins

**The duplicated text from Gojgic-Cvijovic et al., 2018. Revised to Line 384-391**

The pH changes during fermentation on sucrose and on molasses media were compared (S1 Fig(B), S2 Fig) (B). In sucrose medium, a rapid drop of pH occurred during the first 24 h due to acid formation, and afterwards, the pH remained stable in the range of 5.0 - 5.2 (with an initial sucrose concentration of 50 g L^-1^) or 4.3 - 4.8 (with initial sucrose concentrations of 100, 150 and 200 g L^-1^) until the end of fermentation (S1 Fig (B)).

The pH, in molasses medium, was only slightly reduced and ranged from 5.79 to 6.0 throughout the entire fermentation (S2 Fig (B)). Therefore, pH curves were in accordance with previously reported data [49, 75].

**The duplicated text from Han, Watson, 1992. Revised to Line 394-399**

The bacterial conversion of sucrose into levan leads to glucose accumulation, as shown by the decrease in sucrose levels and the accompanying increase in levan and glucose levels in the growth medium. A small amount of fructose was also detected. The sucrose level dropped and levan started to appear during 24 h fermentation; thereafter, the sucrose level rapidly decreased as levan increased. Levan levels peaked at 4 days and reached about 36% when grown on 15% sucrose medium, constituting a 47% theoretical yield based on the available fructose.

**The duplicated text from Yu et al., 2016. Revised to Line 424-436**

The FT-IR spectrum of levan showed a strong, wide band between 3600 cm^-1^ and 3200 cm ^-1^ corresponding to O-H stretching vibrations that exist in polymeric alcohol. Moreover, peaks around 3437, 2928 and 2885 cm ^-1^ corresponding to O-H stretching vibrations and C-H stretching and bending vibrations indicated the existence of polysaccharide. The bending vibration of O-H associated with an intense peak around 1651 cm^-1^ also indicated the presence of bound water.

The stretching vibrations of C-O-C in pyranose or furanose were attributed to peaks around 1126 and 1014 cm^-1^ , while peaks around 922 and 806 cm^-1^ were associated with the symmetrical stretching vibration of furanose and the bending vibration of D-type C-H bonds present in furanose, respectively. Thus, typical signals from furanose were completely different from the signals of pyranose, enabling these two components to be easily distinguished. The existence of C-O-C signals was in accordance with this result. Therefore preliminary assessment of EPS structure showed that it was composed mostly from a D-furanose with no substituent groups.

**The duplicated text from Gojgic-Cvijovic et al., 2018. Revised to Line 472-475**

Levans from the most-studied Gram positive bacterial genera, *Bacillus* and *Paenibacillus,* have molecular weights in the 10^4^ to 10^9^ Da range [71, 72], while levan from *B. licheniformis* 8-37-0-1 has a molecular weight of 2.826 x10^4^ Da [73]. However, the levan produced by *Kozakia baliensis* has an exceptionally high molecular weight of 2.466 x 10^9^ Da [70].

**The duplicated text from Xu et al., 2016. Revised to Line 476-479**

The molecular weights of microbial levans are quite variable depending on the specific producer and the cultivation conditions [74]. For example *B. subtilis* (natto) produced both low (11 kDa) and high (1800 kDa) molecular weight levans. However, *B. polymyxa* (NRRLB-18475) produced only very high molecular weight levan of approximately 2 × 10^6^ Da [71, 75].

**The duplicated text from Gojgic-Cvijovic et al., 2018. Revised to Line 492-495**

Indeed, similar effect was previously reported. Relatively high molecular weight levan (range > 10^6^ Da) is produced in sugar beet molasses-based media with 200 g L^-1^ total sucrose, while relatively lower molecular weight levan (in the range of 10^5^ Da) is produced in medium with high concentrations (400 g L^-1^) of sucrose [49].

**The duplicated text from Revin et al., 2020. Revised to Line 500-509**

Samples used for structural analysis were prepared by freeze-drying. The SEM showed that the EPS surface has complex petal morphology with rounded macropores (Fig 9). The structure of the sample was similar to that of an aerogel, although it is known that levan cannot be gelled without a surface modification. Aerogels are known as highly porous materials with a huge internal surface area [77]. Two methods usually applied to form aerogels are (1) supercritical drying or (2) freeze-drying. In both cases, the structure of the material is preserved during drying.

An analysis of the sample composition was performed using energy-dispersive X-ray spectroscopy (EDX) and the elements present in the sample were analyzed by (**S5 Fig**). The results demonstrated strong C and O signals and allowed the identification of 57.49% carbon and 41.77% oxygen corresponding to groups present in the levan structure

**The duplicated text from Revin et al., 2020.** **Revised to Line 512-519**

The stability and longevity of polymers are usually measured by thermogravimetric analysis. One important characteristic of EPS is its thermostability, especially for high-temperature applications. Indeed, levan can be fully thermally processed through molding and extrusion methods [4]. Therefore, we conducted a thermogravimetric study of the EPSs produced by *P. polymyxa* 2020 during growth in sucrose and molasses media (**Fig 10**). The thermal weight loss of the EPS started at 57°C, and when the treatment reached 200 °C, the EPS lost about 15% of its mass. Thermogravimetric analysis demonstrated that levan begin to degrade at 200°C, and by the time the temperature reached 300 °C about 60% of the EPS mass has been lost.

**The duplicated text from Revin et al., 2020. Revised to Line 522-532**

To investigate the effect of levan produced by *P. polymyxa* 2020 from a sucrose and molasses media, cytotoxicity studies were performed to test the proliferation of a mouse fibroblast cell culture L929. The viability of L929 cells was evaluated by the MTT assay (**S4** **Fig**)

All the materials tested showed very low toxicity. No reduced cell viability was seen during incubation with levan produced by *P. polymyxa* 2020 either in a sucrose or a molasses media. Thus, EPS does not inhibit the proliferation of L929 cells. We also observed that the morphology of L929 cells was not affected by levan produced by *P. polymyxa* 2020 in a sucrose and molasses media. The cells were monitored using an inverted optical microscope and the morphologies of untreated L929 cells were similar to those that had been treated with EPS. These results indicate that levan produced by *P. polymyxa* 2020 is a promising source of functional biomaterials for a wide range of cellular applications, including biomedicine.

**The duplicated text from Gojgic-Cvijovic et al., 2019.** **Revised to Line 45-49**

It has received much attention due to its anticancer, anti-oxidant, antibacterial, anti-inflammation, immunomodulating and prebiotic activities as well as many outstanding physicochemical properties such as low intrinsic viscosity, high adhesive strength, high water solubility, film-forming ability and high biocompatibility [2–4].

**Review Comments to the Author
Reviewer 1**

This study reports about isolation of Paenibacillus polymyxa 2020 from wasp honeycombs capable of producing levan. Whole genome sequencing and methylome analysis of P. polymyxa 2020 were performed and bioinformatic analysis identified putative levan synthetic operon. sacC and sacB genes were further cloned and their products identified. To reduce the production cost inexpensive substrate molasses was investigated for EPS production. Manuscript needs following changes to improve it further.

**Authors' response:** Thank you very much for all of your detailed comments and suggestions. We found them quite useful as we approached our revision.
**Comments:**

**1.** Remove word novel from title as many Paenibacillus strains already isolated and reported for different applications.

**Authors' response:** Thank you very much for your recommendation. The word novel was removed from the title.

**2.** In introduction part add information related to different exopolysaccharides, microorganisms, and their applications. Also add information related to market potential of levan and other EPS.

**Authors' response:** Information related to different exopolysaccharides and microorganisms was added:

Some of the most used EPSs are xanthan from Xanthomonas campestris, dextran produced by Leuconostoc mesenteroides, Streptococcus sp., Lactobacillus sp., alginate from *Azotobacter* sp., *Pseudomonas* sp*.,* gellanderived from Pseudomonas elodea and Sphingomonas paucimobilis, curdlan from *Alcaligenes faecalis*, hyaluronan from *Streptococcus equi*, pullulan from Aureobasidium pullulans, bacterial cellulose from *Komagataeibacter* sp., and levan from *Bacillus* sp., *Paenibacillus* sp., *Halomonas*sp. etc.

Information related to the market potential of EPS was added:

The importance attached to the EPS market and commercial applications encourages the rapid development of research to source new producers, the isolation and production of EPS, and obtaining novel functional materials for a wide range of applications

**3.** Line 138: Why author decided to use 10% v/v inoculum? Why author didn’t try to optimize various culture conditions for optimum growth and EPS production, as this was a new isolate, and these parameters may help to increase production.

**Authors' response:** We plan to present data on the optimization of the various culture conditions for optimum growth and EPS production (the amount of inoculum, the mixing rate, the degree of aeration, etc.), including during the scaling process in bioreactors of various volumes, in the next article due to the large volume of this article.

**4**.What are other components present in EPS? As EPS is composed of carbohydrates, proteins, organic acids etc.

**Authors' response:** The Fourier transform infrared (FT-IR) and nuclear magnetic resonance (NMR) spectra demonstrate that the EPS is a linear β-(2→6)-linked fructan (levan). No other components were found in the EPS. This is also confirmed by the results of SEM-EDX analysis of samples (oxygen - 41.77%, carbon – 57, 49%) (Supporting Information S5Fig).

**5**.Line 339: P. polymyxa 2020 showed a high content of EPS in comparison to other strain at almost same concentration of sucrose. Compare in terms of culture conditions also (pH and T) used in this study and other study to provide better information to readers.

**Authors' response:** A comparison of culture conditions has been added.

**6.** Provide figure number to all the figures.

**Authors' response:** Figure number are provided for all the figures.

**7.** Remove grid lines from the figures

**Authors' response:** Grid lines are removed from the figures.

**8.** Line 99: C/N and pH plays important role in EPS production, while in this study no such experiment included. Need to perform experiment and include results.

**Authors' response:** We plan to present data on the optimization of the various culture conditions for optimum growth and EPS production (the amount of inoculum, the mixing rate, the degree of aeration, etc.), including during the scaling process in bioreactors of various volumes, in the next article due to the large volume of this article. Information about change in pH during growth was added (S1 Fig, S2 Fig).

**Reviewer 2**

Thank you very much for all of your detailed comments and suggestions. We found them quite useful as we approached our revision.
**Comments:**
The paper is interesting in describing a new strain with relevant biosynthetic pathways to exopoysaccharide production, and the ability to achieve high titer of EPS. The authors run a wide array of characterization experiments, from a biomolecular and biological side to a more chemical-structure analyses.
However the manuscript fails to correctly describe in detail the experimental part.
I think there is a need to improve the methodologies section few examples are following:
Improvement of fermentation descriptions, give detains on growth.

**Authors' response:** Information about growth and change in pH during growth was added (S1 Fig, S2 Fig).

explain how they can purify and isolate EPS with such a simple 2 step process (comparison to other EPS, contamination of polysaccharides from teh media...etc.)

**Authors' response:** The Fourier transform infrared (FT-IR) and nuclear magnetic resonance (NMR) spectra demonstrate that the EPS is a linear β-(2→6)-linked fructan (levan). No other components were found in the EPS. This is also confirmed by the results of SEM-EDX analysis of samples (oxygen - 41.77%, carbon – 57, 49%) (Supporting Information FigS5).

Clarify why they use high temperature drying

**Authors' response:** Samples used for structural analysis were prepared by freeze-drying.

improve SEM analyses description

**Authors' response:** SEM analyses description was improved.

compared their limitation in NMR to eventually previously reported spectroscopic analyses of heteropolysaccarides like hyaluronic acid, or other GAGs.;

**Authors' response:** There are more than 10,000 papers describing the NMR spectroscopic analyses of heteropolysaccharides, and most of them provides unique fingerprinting data. Due to this, comparison of spectra to those of other polysaccharides is beyond the scope of this study. The comparative analysis of these data lies behind the usage of automated matching and subsequent manual verification of spectroscopic observables, as described in the manuscript (lines 438 PONE-S-20-50060.pdf, p19 in the revised manuscript, Table 1). To clarify the NMR analysis we refactored the NMR section (lines 413-450 in PONE-S-20-50060.pdf, lines 414-467 in the revised manuscript).

Explain the meaning of the cytotoxicity study with respect to a proposed application;

**Authors' response:** These results showed that levan produced by *P. polymyxa* 2020 is a promising candidate for obtaining functional biomaterials for a wide range of applications, including biomedicine. Therefore, it is non-toxic for cell culture applications.

e) describe the specific interest in the molecular determinant of the biosynthetic pathways, since they report already a very high production (and productivity) when compared to other microbial polysaccharides.
**Authors' response:** We plan to study the biosynthetic pathway in more detail in the future.

Minor points are also highlighted below: The shake flasks growth, although a simple and routine approach should be better described here (biomass density achieved, growth curve, or any othe information like change in pH during growth.

**Authors' response:** Information about biomass and change in pH during growth was added (S1 Fig, S2 Fig).

If the process is a fully aerobic one it seems that the inoculums of 100 mL medium in a 250 mL flask is not ensuring a sound gas distribution, so is limiting oxygen transfer, this especially in relation to the increase n velocity during growth.

**Authors' response:** In our work, a fairly high mixing speed was used -250 rpm.

In Figure 2 the production of levan is presented without any information on the biomass growth. Since the authors are using a very high sugar content (either a disaccharide or a complex mixtures ..also containing oligo saccharides) the osmotic pressure will be different in the various condition and thus I will expect a difference in growth rate and then of Levan production.

**Authors' response:** Information about biomass growth was added (S1 Fig, S2 Fig).

The reference at time zero (for zero amount of levan) was quantified on the medium of this was an assumption of the authors? Please specify.

**Authors' response:** The concentration of levan was measured right after addition of inoculum

The downstream procedure described is very simple, I argue it is very difficult to separate pure EPS only using precipitation and dialysis, did the author apply the same procedure to the fermentation broth to analyze similar residues directly coming from the complex components (e.g. yeast extract or other)

**Authors' response:** We applied the same procedure to the fermentation broth. However, no precipitation was observed. Repeated precipitation and dissolution in water was performed to purify the levan. The Fourier transform infrared (FT-IR) and nuclear magnetic resonance (NMR) spectra demonstrate that the EPS is a linear β-(2→6)-linked fructan (levan). No other components were found in the EPS. This is also confirmed by the results of SEM-EDX analysis of samples. The results showed strong signals of C and O (oxygen - 41.77%, carbon – 57, 49%) (Supporting Information S5 Fig).

Fig 10. SEM images of EPS of P. polymyxa 2020 at a various magnification- please improve description in the legend

**Authors' response:** SEM image and description in the legend were changed.

SEM micrograph should be better explained in the text (how the films were obtained, and with procedeure before metalization. It is not clear why a porosity should be found if not for very low molecular weight components (like salts) that may leave the film if washed. The authors should clarify this point

**Authors' response:** SEM analyses description was changed:

Samples used for structural analysis were prepared by freeze-drying. The SEM showed that the EPS surface has complex petal morphology with rounded macropores (Fig 9). The structure of the sample was similar to that of an aerogel, although it is known that levan cannot be gelled without a surface modification. Aerogels are known as highly porous materials with a huge internal surface area [77]. Two methods usually applied to form aerogels are (1) supercritical drying or (2) freeze-drying. In both cases, the structure of the material is preserved during drying.

An analysis of the sample composition was performed using energy-dispersive X-ray spectroscopy (EDX) and the elements present in the sample were analyzed by (**S5 Fig**). The results demonstrated strong C and O signals and allowed the identification of 57.49% carbon and 41.77% oxygen corresponding to groups present in the levan structure

Figure 12 is very confusing, and low quality

**Authors' response: Fig12** has been replaced with a higher quality **Fig11**, originally submitted gel image for panel C was lack of protein markers, therefore it was replaced with a new image. As a request of PlosOne we provide original unadjusted and uncropped images as supporting **S7 Fig**.
