# Supplementary material for: Production and сharacterization of the exopolysaccharide from strain Paenibacillus polymyxa 2020
Source: PLoS One. 2021 Jul 6;16(7):e0253482. doi: 10.1371/journal.pone.0253482 (PMC8259973; doi:10.1371/journal.pone.0253482)
Supplement: Attachment — Submitted filename: Rebuttal letter.docx [file pone.0253482.s010.docx]

Dear Dharam Singh
Academic Editor
PLOS ONE

*Thanks for giving us the opportunity to fix* *the English usage and inelegant structure of the manuscript. Dr. Roberts, a native English speaker has gone through the manuscript carefully and we hope that it now conforms to PLoS ONE standards. The small corrections raised in points 2-4 have also been corrected.  We apologize for the writing, which reflected an initial draft in Russian, translation to English by a bi-lingual author and a less than careful earlier check by Dr. Roberts.  Hopefully, the current version will prove acceptable.*

1.Writing part is poorly managed through the manuscript. Basically, each paragraph should be arranged as a unit that should be in coherent with another para. But, if you see text has been arranged as small (5-6 lines) distributed text. See lines 65-75. They can be come under single para. Similar is the problem throughout the introduction, materials and methods section, results and discussion. To refer few of them as lines 353-394, 414-424, 478-491 etc..

*Dr. Roberts, a native English speaker has gone through the manuscript carefully and we hope that it now conforms to PLoS ONE standards*

2. Supplementary S6: correct the concentration from 0,31 to 0.31 mg/ml. Similarly for other values too.

*S6Fig was corrected*

3. S7 Fig. is not labeled. Position of Protein marker is not defined.

*S7Fig was corrected, position of Protein marker was added*

4. What is the PI of cloned proteins? May be the negative charge of proteins have led to slow migration in the gel in S7Fig.

*Estimated isoelectric point of SacB protein is 5.194 (-10.741 charge at pH7.0) and isoelectric point of SacC protein is 8.869 (7.714 charge at pH7.0) (*[*https://web.expasy.org/compute_pi/*](https://web.expasy.org/compute_pi/)*).*

5. Besides, there are still several mistakes through the manuscript that need to be corrected.

*Please see response to comment1*

Sincerely,

Alexey Fomenkov
